# The Prevalence and Psychosocial Factors of Problematic Smartphone Use Among Chinese College Students: A Three-Wave Longitudinal Study

**DOI:** 10.3389/fpsyg.2022.877277

**Published:** 2022-04-05

**Authors:** Anqi Wang, Zhen Wang, Ya Zhu, Xuliang Shi

**Affiliations:** ^1^College of Education, Hebei University, Baoding, China; ^2^School of Public Administration, Guangzhou Xinhua University, Dongguan, China; ^3^Center for Mental Health Education and Counseling, Guangdong University of Science and Technology, Dongguan, China

**Keywords:** problematic smartphone use, longitudinal study, prevalence, psychosocial factors, college students

## Abstract

Problematic smartphone use (PSU) in college students has been a major public health concern in modern society, which may also lead to adverse health outcomes. Using a three-wave longitudinal study design, the current study aimed to examine the prevalence and psychosocial factors of PSU in a large sample of Chinese college students. The data used in this study was obtained from an ongoing longitudinal study in Guangdong, China. In the current study, a total of 7,434 freshmen and sophomores who completed the first three surveys were included. Self-administered questionnaires were used to assess PSU, possible social anxiety disorders, depressive symptoms, loneliness, family conflicts, academic stress, and some demographic characteristics. Generalized estimating equation (GEE) models were performed to determine the risk factors associated with PSU. The results showed that 65.8, 58.1, and 52.8% of college students reported PSU at three waves, with an apparent downward trend. Female students reported higher prevalence rates of PSU than males. Depressive symptoms, possible social anxiety disorders, loneliness, family conflicts, and high academic pressure were important risk factors for PSU. Early intervention and identification of those who show signs of PSU may prevent the development of maladaptive coping responses and addictive behaviors, so as to prevent future negative psychosocial consequences.

## Introduction

With the advent of the Internet era, smartphones have been updated rapidly, which caused drastic changes in our daily life. The COVID-19 pandemic has also made most people incapable of living without smartphones and network platforms during home quarantine. Because of the extremely large population base, China has the most smartphone users worldwide. The rapid adoption of electronic devices like smartphones has been attributed to their portability and various functions such as online shopping, electronic payment, easy access to social media platforms, mobile games, and navigation systems (Chen et al., 2017). Therefore, smartphone is not only a communication tool, but also a real-time information provider and a powerful portable computer (Long et al., 2016). Despite the positive aspects associated with smartphone use, there is still growing evidence indicating that smartphone overuse has adverse effects on interpersonal relations, physical and mental health (Xie et al., 2018; Grant et al., 2019; Winkler et al., 2020).

Various terms are given for different patterns of smartphone overuse, such as “problematic smartphone use,” “smartphone addiction,” “mobile phone addiction,” and “excessive smartphone use” (Elhai et al., 2017). In our current study, we use the description of “problematic smartphone use (PSU),” which has been defined as a cognitive, emotional and behavioral pattern of excessive use of smartphones that leads to negative consequences in daily life (Billieux, 2012). PSU is considered a potential behavioral addiction, and it consists of four main components: compulsive behaviors like checking for messages over and over again; tolerance, more prolonged and more intense use; withdrawal, feeling distressed without the smartphone; and functional impairment, interference with social activities (Lin et al., 2014, 2016).

College students are in a critical period of transition from adolescence to early adulthood, yet their psychological and cerebral development is not totally completed. As young people, they are also among the age groups targeted by communication technologies. Therefore, compared with older social groups, college students were shown to be more vulnerable to smartphones. Moreover, most college students are grown up surrounded by various electronic devices like smartphones, which have been integrated into their lifestyle and identity (Long et al., 2016). They are also the most interested in possessing smartphones on which they spend time and dedicate much of their energy (Aljomaa et al., 2016). Based on the above reasons, college students are most likely to become dependent on smartphones, which may lead to addictive behaviors. However, limited literature explored the prevalence and psychosocial factors of PSU by using a longitudinal study design with a large sample of Chinese college students.

It is difficult to determine the prevalence of PSU due to the lack of consistency in possible diagnostic criteria, economic and cultural differences between different countries, and the survey of highly restricted samples. Currently, some cross-sectional studies have reported a high prevalence of PSU in adolescents and adults (Long et al., 2016; Lee and Lee, 2017; Nahas et al., 2018; Buctot et al., 2020; Okasha et al., 2021). In a systematic review and meta-analysis, researchers examined the prevalence of PSU among children and young people, and the results showed that the prevalence was between 10 and 30%, and the median was 23.3% (Sohn et al., 2019). As for Chinese college students, Wang and Zhang (2015) surveyed 4,000 Chinese college students and found that the prevalence rate of PSU was 37.9%. Another study conducted by Long et al. (2016) found that 21.3% of university students were addicted to smartphones. Due to this high prevalence, it is necessary to explore what kind of factors will have a significant impact on PSU.

Some previous studies have demonstrated that socio-demographic factors (e.g., gender, age, residence), family and social environmental factors (e.g., family relation, peer relationship, school atmosphere), and psychological factors (e.g., depression, anxiety, loneliness, stress) had an important effect on PSU (Luk et al., 2018; Elhai et al., 2019; Fischer-Grote et al., 2019; Jin Jeong et al., 2020; Ouyang et al., 2020). However, these studies were limited to cross-sectional nature, small sample sizes, and did not statistically control for potential confounding factors. Only a few longitudinal studies have explored the risk factors of PSU. For example, using a three-wave longitudinal design, Yuan et al. (2021) found that depression severity at baseline was significantly related to subsequent PSU. Furthermore, in a recent longitudinal study of Chinese college students pre and during COVID-19, Yang et al. (2021) found a significant prospective association between loneliness and PSU.

Numerous theories have tried to explain the psychological mechanisms underlying PSU. The Compensatory Internet Use Theory (CIUT) (Kardefelt-Winther, 2014) posits that negative life situations can give rise to different motivations, then drive problematic technology use. In other words, excessive internet use could be a maladaptive coping strategy to escape from negative affective states (Rozgonjuk et al., 2018; Della Vedova et al., 2022). It has been found that individuals experiencing stress or negative events often seek technology use as a way to alleviate emotional distress. Therefore, people with psychopathological symptoms like depression and anxiety may use the internet as compensation for reality. Another theoretical framework is the Interaction of Person-Affect-Cognition-Execution (I-PACE) model (Brand et al., 2019). This model involves a complex set of variables, which can be the moderator or mediator between individual characteristics and specific Internet-use disorders. There are three levels in this model: (1) a person’s core characteristics (P-component); (2) cognitive and affective factors (A and C-components), and (3) executive functions (E-component). This model suggests that personal traits or cognitive and affective factors may influence PSU. Based on the above-mentioned theories and empirical studies, using a longitudinal study design, the current study was conducted to examine the prevalence and psychosocial factors of PSU in a large sample of Chinese college students. We hypothesized that the psychopathological symptoms (social anxiety, depression, and loneliness) and negative life situations (academic stress, family conflict) could predict later PSU.

## Materials and Methods

### Participants and Procedures

The data used in this study was obtained from an ongoing longitudinal study in Guangdong, China. Detailed sampling and data collection have been described in our previous study (Jiang et al., 2021; Shi et al., 2021). In brief, participants were sampled from three universities of Guangdong, with consideration of prior study collaboration, convenience and budget. At baseline (T_1_), a total of 11,740 freshmen and sophomores completed the questionnaire after excluding 964 invalid questionnaires. Students who did not respond or responded in less than fifteen minutes were excluded. These participants were assessed again after 6 (T_2_) and 18 (T_3_) months. In the current study, a total of 7,434 participants who completed three surveys were included. The main reasons for the attrition are that students asked for leave on the day of assessment and senior students graduated. Chi-square tests were used to compare demographic characteristics at baseline for participants who completed three surveys with those who did not. The results found that males (χ^2^ = 75.76, *df* = 1, *p* < 0.001) were more likely to drop out.

For data collection, a self-administered, structured questionnaire in the Chinese language was distributed to participants through an online questionnaire platform during regular school hours. This survey was delivered with the help of a group of well-trained and experienced teachers and graduate students and all participants were required to read the instructions carefully and they were informed that their responses were voluntary and confidential. The process of the whole survey was lasted approximately 30–40 min. All participants were informed that they could withdraw at any time if they felt uncomfortable. We obtained permission to conduct the study from the principals in the target schools and obtained written informed consent from the participating students before the survey. The study was approved by the Research Ethics Committee of the corresponding author’s institution.

## Measures

### Problematic Smartphone Use

Problematic smartphone use was evaluated by the Smartphone Addiction Scale short version (SAS-SV) (Kwon et al., 2013), which consists of 10 items (e.g., “*Missing planned work due to smartphone use*”). Respondents rated each item on a 6-point scale ranging from 1 = *strongly disagree* to 6 = *strongly agree*. The total score ranges from 10 to 60, with higher scores indicating a high degree of smartphone use. SAS-SV cut-off scores of ≥31 for males and ≥33 for females were used as proposed by the scale developers. This scale has been demonstrated good reliability and validity in Chinese adults (Luk et al., 2018). In this study, the Cronbach’s alpha values were 0.89, 0.89, and 0.92 at T_1_, T_2_, and T_3_, respectively.

### Possible Social Anxiety Disorders

Possible social anxiety disorders (SAD) were measured with the subscale of the Screen for Adult Anxiety Related Disorders (SCAARED; Angulo et al., 2017). This subscale consists of seven items (e.g., “*I don’t like to be with people I don’t know well*”) that are rated on a 3-point scale (0 = *not true or hardly ever true*, 1 = *somewhat true or sometimes true*, and 2 = *very true or often true*), and the total score ranges from 0 to 14. A higher total score indicates a higher level of social anxiety. This scale has demonstrated good reliability and validity among Chinese adults (Chen et al., 2021). A cutoff score of 7 has been recommended for identifying possible SAD (Angulo et al., 2017). In this study, the Cronbach’s alpha values were 0.86, 0.87, and 0.89 at T_1_, T_2_, and T_3_, respectively.

### Depressive Symptoms

Patient Health Questionnaire (PHQ-9) was used to assess the severity of depressive symptoms over the past 2 weeks (Kroenke and Spitzer, 2002). The PHQ-9 consists of nine statements assessed on a 4-point scale from 0 (*not at all*) to 3 (*nearly every day*). The total score ranges from 0 to 27, with a higher score indicating a higher level of depression. The Chinese version of PHQ-9 had been demonstrated good psychometric properties in the general population (Wang et al., 2014). A cutoff score of 10 has been recommended for identifying probable depression. In this study, the Cronbach’s alpha values were 0.86, 0.86 and 0.89 at T_1_, T_2_ and T_3_, respectively.

### Loneliness

The Chinese version of the ULS-8 was used to measure the level of loneliness (Hays and DiMatteo, 1987; Wu and Yao, 2008), which is the short version of UCLA (University of California Los Angeles Loneliness Scale). This scale contains eight items (e.g., “*People are around me but not with me*”) indexed on a 4-point scale ranging from 1 (*never*) to 4 (*always*). The total score ranges from 8 to 32, with higher scores indicating a higher degree of loneliness. ULS-8 has been widely used in China and demonstrated excellent psychometric properties. In this study, the Cronbach’s alpha values were 0.83, 0.85, and 0.84 at T_1_, T_2_, and T_3_, respectively.

### Family Conflicts

Family conflicts were measured with the subscale of the Family Environment Scale-Chinese Version (FES-CV) (Phillips, 1999). This subscale consists of nine self-report items (e.g., “*Family members often blame and criticize each other*”). In the original scale, all items were answered with “yes” or “no.” In this study, we adapted it into a 4-point scale from 1 (*never*) to 4 (*always*). After reversing three items, the total score was calculated by adding up nine items. The total score ranges from 9 to 36, with a higher score demonstrating a higher level of intra-family conflict. The Chinese version of FES-CV has shown excellent reliability and validity among Chinese people (Phillips, 1999). In this study, the Cronbach’s alpha value was 0.77 at T_1_.

### Academic Stress

Academic stress was measured by one single self-reported item: “How is your current academic stress”. This item was evaluated on a 3-point scale from 1 = *low or lower*, 2 = *general*, to 3 = *high or higher*.

### Covariates

Previous studies have found that some individual (e.g., age, gender, siblings, and residence) and family-related factors (e.g., parents’ education) were associated with problematic smartphone use (Kwon et al., 2013; Aktürk et al., 2018; Luk et al., 2018). Based on the above studies, we chose these variables as possible covariates.

### Statistical Analyses

First, descriptive analyses were conducted on the prevalence of PSU at three different time points. To determine whether there were any statistically significant differences in demographics between students who had PSU and those without PSU at baseline, we performed Chi-square tests for categorical variables and Student t-tests for continuous variables. Second, descriptive statistics (means and standard deviations) and the correlation matrix among main variables are shown in Table 2. Third, for the convenience of data analysis, some variables were recoded. According to the clinical cutoff value, depression and social anxiety were recoded as “yes” versus “no.” Since there was no clinical cutoff value, the total score of loneliness and family conflict were recoded into three categories (“mild,” “moderate,” and “severe”), with mild and severe categories defined by M-SD and M + SD, respectively. In order to identify the specific risk factors of PSU among college students, generalized estimation equation (GEE) models with a logit link function having robust variances were used to examine the relationship between the selected variables and PSU (Zeger and Liang, 1986), and the working correlation matrix was modeled as independent. In the GEE models, demographics, family conflicts and academic stress at T_1_ were added as time-invariant variables, and possible SAD, depressive symptoms and loneliness were modeled as time-varying variables. Odds ratios (OR) and 95% CIs were reported to demonstrate the associations of risk factors with PSU compared with no PSU. Specifically, a total of three models were examined. Model 1 is the crude model without adjusting for any covariates. Model 2 tested whether PSU was predicted by previous psychosocial factors after adjusting for gender, only child and parents’ education level. Based on Model 2, Model 3 further controlled for all time-varying and time-invariant variables. All analyses were conducted with IBM SPSS Version 23.0, with a significant α threshold of 0.05 (two-tailed).

**TABLE 1:** Demographic characteristics of the sample at baseline (*N* = 7,434).

Variable^a^	Overall *n* (%)	Problematic smartphone use		χ*^2^*/*t*	*p*
		No (%)	Yes (%)		
Age, *M* (SD)	19.67 (1.15)	19.70 (1.11)	19.65 (1.16)	1.83	0.067
Gender	7,434			20.66	**<0.001**
Male	3,848 (51.8)	1,401 (36.7)	2,437 (63.3)		
Female	3,586 (48.2)	1,125 (31.8)	2,447 (68.2)		
Only child	7,407			6.05	**0.014**
Yes	1452 (19.6)	535 (36.8)	917 (63.2)		
No	5955 (80.4)	1,991 (33.4)	3,964 (66.6)		
Residence	7,407			0.22	0.639
Urban	3,359 (45.3)	1,136 (33.8)	2,223 (66.1)		
Rural	4,048 (54.7)	1,390 (34.3)	2,658 (65.7)		
Father education	7,406			8.18	**0.017**
Middle school	4,484 (60.5)	1,497 (33.4)	2,987 (66.6)		
High school	1,874 (25.3)	631 (33.7)	1,243 (66.3)		
College or above	1,048 (14.2)	398 (38.0)	650 (62.0)		
Mother education	7,407			14.56	0.001
Middle school	5,310 (71.7)	1,748 (32.9)	3,562 (67.1)		
High school	1,413 (19.1)	507 (35.9)	906 (64.1)		
College or above	684 (9.2)	271 (39.6)	413 (60.4)		

*^a^Number of participants differed from total N = 7,434 due to missing data; M, mean; SD, standard deviation. Bolded values indicate the signficant results.*

**TABLE 2:** Zero-order correlations among main variables.

Variables	1	2	3	4	5	6	7	8	9	10	11	12	13	14
1. PSU (T_1_)	1													
2. PSU (T_2_)	0.62	1												
3. PSU (T_3_)	0.52	0.62	1											
4. Possible SAD (T_1_)	0.38	0.33	0.32	1										
5. Possible SAD (T_2_)	0.30	0.41	0.34	0.68	1									
6. Possible SAD (T_3_)	0.29	0.35	0.49	0.57	0.65	1								
7. Depressive symptoms (T_1_)	0.43	0.36	0.34	0.50	0.37	0.35	1							
8. Depressive symptoms (T_2_)	0.35	0.45	0.37	0.37	0.49	0.39	0.58	1						
9. Depressive symptoms (T_3_)	0.31	0.36	0.49	0.34	0.38	0.58	0.49	0.57	1					
10. Loneliness (T_1_)	0.38	0.35	0.34	0.55	0.47	0.42	0.61	0.48	0.43	1				
11. Loneliness (T_2_)	0.32	0.43	0.37	0.45	0.55	0.45	0.46	0.58	0.46	0.66	1			
12. Loneliness (T_3_)	0.29	0.35	0.47	0.40	0.43	0.55	0.42	0.45	0.58	0.58	0.63	1		
13. Family conflicts	0.24	0.21	0.20	0.21	0.17	0.18	0.33	0.29	0.26	0.33	0.28	0.26	1	
14. Academic stress	0.13	0.12	0.12	0.14	0.11	0.09	0.23	0.19	0.16	0.20	0.16	0.15	0.13	1
M	34.47	32.67	31.12	5.28	5.03	4.21	5.51	4.94	4.08	15.53	15.88	15.09	17.56	3.28
SD	9.77	10.17	10.75	3.37	3.30	3.34	3.71	3.65	3.70	4.61	4.65	4.57	4.45	0.73

*PSU, Problematic smartphone use; SAD, Social anxiety disorders; All correlation coefficients are significant at 0.001 (two-tailed).*

## Results

### Sample Characteristics

Demographic information and differences between those with and without PSU are shown in Table 1. At baseline, the mean age of participants was 19.67 years (SD = 1.15). In the total sample, 51.8% were males and 80.4% were not the only child in their families. Regarding parents’ education level, 60.5% of fathers and 71.7% of mothers have less than or equal to 9 years of education. As for residence, more than half of the students (54.6%) were from rural areas. In addition, the results of Chi-square tests and *t*-tests showed that gender, siblings, and parents’ education were associated with PSU.

### The Prevalence of Problematic Smartphone Use

**FIGURE 1:**
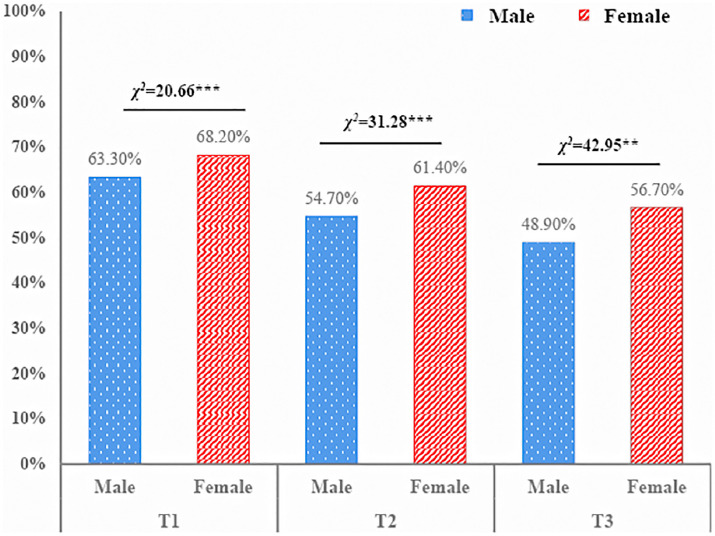
The prevalence of problematic smartphone use among male and female college students.

Overall, 65.8, 58.1, and 52.8% of college students reported PSU, showing an obvious downward trend. As for different genders (see Figure 1), female students reported higher prevalence rates of PSU than males (T_1_: χ^2^ = 20.66, *p* < 0.001; T_2_: χ^2^ = 31.28, *p* < 0.001; T_3_: χ^2^ = 42.95, *p* < 0.001).

### The Psychosocial Factors of Problematic Smartphone Use

As shown in Table 2, PSU was significantly correlated with possible SAD, depressive symptoms, loneliness, family conflicts and academic stress (all *p* < 0.001). Generalized estimating equation (GEE) model was used to examine the risk factors for smartphone addiction (see Table 3). In adjusted model 2, the results suggested that students with possible SAD (AOR = 2.45), depressive symptoms (AOR = 1.59), loneliness (AORs = 2.58–5.31), high academic pressure (AOR = 1.21), and those who suffered from family conflicts (AORs = 1.35–1.63) were at higher risk of PSU.

**TABLE 3:** The psychosocial factors of problematic smartphone use (PSU) using a generalized estimating equation (GEE) model.

Variables	Crude model			Adjusted Model 1^a^			Adjusted Model 2^b^		
	OR	95% CI	*p*	OR	95% CI	*p*	OR	95% CI	*p*
**Possible SAD^c^**									
No	1			1			1		
Yes	3.69	3.21–4.49	<0.001	3.64	3.36–3.93	<0.001	2.45	2.26–2.66	<0.001
**Depressive symptoms^c^**									
No	1			1			1		
Yes	3.79	3.21–4.49	<0.001	3.75	3.17–4.44	<0.001	1.59	1.33–1.91	<0.001
**Loneliness^c^**									
Mild	1			1			1		
Moderate	3.36	3.08–3.66	<0.001	3.29	3.02–3.59	<0.001	2.58	2.36–2.81	<0.001
Severe	10.68	9.38–12.16	<0.001	10.46	9.19-11.92	<0.001	5.31	4.64–6.09	<0.001
**Family conflicts**									
Mild	1			1			1		
Moderate	1.66	1.49–1.84	<0.001	1.62	1.45–1.80	<0.001	1.35	1.21–1.50	<0.001
Severe	2.80	2.41–3.24	<0.001	2.67	2.30–3.10	<0.001	1.63	1.40–1.90	<0.001
**Academic stress**									
Low or lower	1			1			1		
General	1.25		<0.001	1.22	1.07–1.40	<0.01	1.02	0.89–1.17	
High or higher	1.75		<0.001	1.71	1.49-1.97	<0.001	1.21	1.04–1.39	<0.01

*SAD, Social anxiety disorders; OR, odds ratio; 95% CI, 95% confidence interval.*

*^a^Adjusted for all of the significant variables listed in Table 1.*

*^b^Adjusted for all of the significant variables listed in Table 1 and other variables listed in Table 3.*

*^c^Time-varying covariates.*

## Discussion

Although previous studies have examined the psychosocial factors of PSU, most of them were limited to small sample sizes and cross-sectional studies. To our knowledge, this study was the first longitudinal study using GEE model to explore the psychosocial factors of PSU in a large sample of Chinese college students. In our study, the main findings include: (1) the prevalence rate of PSU at three time points is high, but it shows an obvious downward trend with the passage of time; (2) compared with male college students, female college students have a higher prevalence rate of PSU; (3) possible SAD, depressive symptoms, loneliness, family conflicts, and high academic pressure are important risk factors for PSU in college students. These findings may be important and helpful for developing targeted interventions to reduce PSU among college students.

### The Prevalence of Problematic Smartphone Use

The prevalence of PSU observed in our sample was higher than that of young people in other countries such as Switzerland (16.9%) (Haug et al., 2015), Japan (26.4%) (Tateno et al., 2019), United Kingdom (38.9%) (Sohn et al., 2021), Spain (12.5%), and Belgium (21.5%) (Lopez-Fernandez, 2017) using the SAS-SV. However, similar to our study, Okasha et al. (2021) investigated 1,380 Egyptian university students and found that the prevalence rate of PSU was 59.6%. This discrepancy across studies may be due to the economic differences between different countries. With the rapid development of the economy, China has become the largest market for smartphones in recent years, and the market continues to grow at an astonishing pace. As a result, China has the most smartphone users worldwide due to its extremely large population base, especially among college students. Cultural differences may also contribute to the discrepancy. In a cross-cultural study, Lachmann et al. (2018) found that Chinese teenagers (63.6%) had a markedly higher PSU rate than that of Germany (7.5%). Note that the criteria for the diagnosis of PSU were established on Korean adolescents (Kwon et al., 2013), which may be less applicable to young adults. Moreover, a decline in the prevalence of PSU was found in our study. After entering the university, freshmen are presented with a unique set of challenges, stressors, and experiences. In order to alleviate the anxiety caused by maladjustment, they may use smartphones more frequently. As they gradually adjust to college life, their dependence on smartphone may decrease and maintain a stable level. Gender-specific analyses showed that female students had a higher risk of PSU, which was in accordance with some previous studies (Kwon et al., 2013; Demirci et al., 2015; Luk et al., 2018). This difference may be related to the usage pattern of smartphone (Demirci et al., 2015; Luk et al., 2018). Females were more likely to use smartphone to communicate with others through social networking services, while for males, a more diversified type of usage was observed (De-Sola Gutiérrez et al., 2016). Further studies are warranted to unravel the inconsistent prevalence of PSU in males and females.

### Psychosocial Factors Associated With Problematic Smartphone Use

Firstly, our study found that possible SAD and depressive symptoms were statistically significant risk factors for PSU, which is consistent with most previous studies (Enez Darcin et al., 2016; Elhai et al., 2017, 2019; Okasha et al., 2021). According to the model of compensatory internet use proposed by Kardefelt-Winther (2014), electronic devices such as smartphones can be viewed as an avoidance-coping strategy, which provides a feasible substitution of discomforting face-to-face contact with the social situations for individuals. In other words, smartphone can be used as a tool to withdraw from negative affections. When individuals are used to using smartphones to cope with emotional problems (e.g., depression or anxiety), other alternative coping styles (e.g., social support, health-promoting behavior) will be diminished, which in turn lead to PSU. Studies have also found that depressed individuals were more likely to use social media in order to avoid social interaction (Kim et al., 2015; Aljomaa et al., 2016), and avoidance coping responses mediated the relationship between depression and Internet addiction (McNicol and Thorsteinsson, 2017). Moreover, virtual socialization can alleviate the fear or worry of communication in reality, which provides the opportunity to feel free and to behave without the perception of pressure in people with social anxiety (Enez Darcin et al., 2016).

Secondly, our results found that loneliness was an important risk factor for PSU, suggesting that individuals who feel lonely are more likely to overuse their mobile phones. This finding was consistent with previous studies in adolescents (Mahapatra, 2019) and young adults (Enez Darcin et al., 2016; Jiang et al., 2018). For example, Mahapatra (2019) surveyed 330 adolescents and found that loneliness was the main antecedent of smartphone addiction. Another study selected international students in China as participants, and found that international students were more vulnerable to smartphone addiction after experiencing severe loneliness (Jiang et al., 2018). PSU might be a self-treatment for people who experience feelings of loneliness, as smartphones can provide them a different form of socialization (Enez Darcin et al., 2016). In order to seek emotional support and satisfaction, individuals with high loneliness often use smartphones to solve or avoid loneliness, and then form a smartphone dependence.

Thirdly, we found that family conflict was associated with the risk for PSU, which indicated that family factors play an important role in smartphone addiction. Previous studies have documented that family communication (Jin Jeong et al., 2020), parent-adolescent conflict, perceived family satisfaction (Yen et al., 2007) and parental neglect (Kwak et al., 2018) are all related to Internet addiction or smartphone addiction. For instance, Yen et al. (2007) have reported that adolescents with higher conflict with parents would refuse to conform to the supervision of parents, including rules set for Internet use. Similarly, another study found that those who experienced domestic violence were at an increased risk for smartphone addiction (Kim et al., 2018). In dysfunctional families, individuals rarely share their true thoughts with their parents, and they may get less emotional support and psychosocial resources, which can increase their negative emotions and loneliness, thus increasing the risk of smartphone addiction.

Finally, we found that high academic stress had a significant positive effect on PSU, and this result was in line with previous studies indicating that an increase in stress level caused an increase in PSU (Chiu, 2014; van Deursen et al., 2015; Gökçearslan et al., 2018). Chiu (2014) surveyed 387 Taiwanese university students and found that family pressure and emotional stress had positive predictive power for smartphone addiction. Young (2007) indicated that individuals would make more impulsive behaviors, including PSU, in order to alleviate emotional tension. In other words, PSU was converted into a coping strategy for alleviating daily pain and tension.

## Strengths and Limitations

The present study had several strengths, including a large sample size, three-wave longitudinal design, and the control for a number of demographic covariates. However, several limitations should be considered in interpreting the results. First, the self-rated nature of the questionnaire might make answers biased based on social desirability. More effective methods (e.g., interview, behavioral measures) should be considered in future studies to reduce the influence of recall bias. Second, this study only reflects the characteristics of college students in Guangdong, China, and the results might not be generalized to other cultures or geographical areas. Given the generalizability of the findings, future studies should replicate these results in other samples. Third, academic stress was assessed by a single item, which may lead to low reliability. It is necessary to use longer and better-structured questionnaires in the future. Finally, we only evaluated the severity of PSU, rather than the contents and patterns of PSU. Thus, future studies should explore more details about PSU.

## Conclusion

In summary, this is the first longitudinal study based on a large sample size to examine the prevalence and psychosocial factors of PSU in Chinese college students. We found that the prevalence of PSU decreased with the passage of time, and female students had a higher risk of PSU. More importantly, the current findings showed that social anxiety, depression, loneliness, family conflict, and academic stress were independent risk predictors of PSU. Our findings highlight the importance of screening and managing PSU. Early intervention and identification of those who show signs of PSU may prevent the development of maladaptive coping responses and addictive behaviors, so as to prevent future negative psychosocial consequences. In addition, the results of this study can also provide some guidance for mental health professionals in the school settings to design cognitive behavioral interventions and prevention programs. In future studies, more psychological factors related to PSU can be examined, and cross-lagged models can be employed to explore the bidirectional relationships among these variables.

## Data Availability Statement

The original contributions presented in the study are included in the article/supplementary material, further inquiries can be directed to the corresponding author.

## Ethics Statement

The studies involving human participants were reviewed and approved by Hebei University. We obtained permission to conduct the study from the principals in the target schools and obtained informed consent from the participating students before the survey. The patients/participants provided their written informed consent to participate in this study.

## Author Contributions

AW performed the statistical analysis and wrote the first draft of the manuscript. ZW and YZ contributed to manuscript revision. XS contributed to conception, design of the study, and manuscript revision. All authors read and approved the final manuscript.

## Conflict of Interest

The authors declare that the research was conducted in the absence of any commercial or financial relationships that could be construed as a potential conflict of interest.

## Publisher’s Note

All claims expressed in this article are solely those of the authors and do not necessarily represent those of their affiliated organizations, or those of the publisher, the editors and the reviewers. Any product that may be evaluated in this article, or claim that may be made by its manufacturer, is not guaranteed or endorsed by the publisher.
